# miR-125a-3p/FUT5-FUT6 axis mediates colorectal cancer cell proliferation, migration, invasion and pathological angiogenesis via PI3K-Akt pathway

**DOI:** 10.1038/cddis.2017.352

**Published:** 2017-08-03

**Authors:** Leilei Liang, Chengshun Gao, Yang Li, Mingming Sun, Jingchao Xu, Huairui Li, Li Jia, Yongfu Zhao

**Affiliations:** 1 Department of General Surgery, The Second Hospital of Dalian Medical University, Dalian, China; 2 Department of Anesthesiology, The Second Hospital of Dalian Medical University, Dalian, China; 3 College of Laboratory Medicine, Dalian Medical University, Dalian, China

## Abstract

The fucosyltransferase (FUT) family produces glycans, a fundamental event in several cancers, including colorectal cancer (CRC). miR-125a-3p is a non-coding RNA that can reduce cell proliferation and migration in cancer. In this study, we explored the levels of miR-125a-3p and FUT expression in human CRC tissues and two human CRC cell lines by qPCR. The results showed that miR-125a-3p, FUT5 and FUT6 are differentially expressed in normal and tumour tissues. On the basis of our previous research, FUT can be regulated by miRNA, which influences the proliferation and invasion of breast and hepatocellular cancer cells. We hypothesised that FUT5 and FUT6 may be regulated by miR-125a-3p. Luciferase reporter analyses were applied to identify potential target genes of miR-125a-3p. A functional study showed that miR-125a-3p overexpression can inhibit the proliferation, migration, invasion and angiogenesis of CRC cells via down-regulating FUT5 and FUT6. In addition, regulating miR-125a-3p, FUT5 or FUT6 expression markedly modulated the activity of the PI3K/Akt signalling pathway, and this effect of FUT5 or FUT6 could be reversed by transfection with miR-125a-3p-mimics. Taken together, our data suggest that both FUT5 and FUT6 can promote the development of CRC via the PI3K/Akt signalling pathway, which is regulated by miR-125a-3p. miR-125a-3p may serve as a predictive biomarker and a potential therapeutic target in CRC treatment.

Colorectal cancer (CRC) is the third leading of death in the world.^1^ Although surgical resection is the best treatment for CRC, many patients fail to carry out operation because of cancer complications.^2^ A better understanding of the biology of CRC is essential for effective treatment methods.^3^ As targeted therapy has been applied in advanced CRC treatment, recent treatments have been greatly enhanced and quality of life has progressed.^4, 5^

The fucosyltransferase (FUT) family is a group of fucosylation synthases that transfer their catalytic fucose from GDP-fucose to oligosaccharides, sugar chains of glycoproteins or glycolipids on the substrate.^6, 7^ Through the inhibition of the biosynthesis of a sugar chain interruption on the surface, the FUT gene is an attractive therapeutic target for therapeutic studies.^8^ This family of three genes (FUT3, FUT5 and FUT6) constitutes a cluster within 1 cM on human chromosome 19p13.3^9, 10^ and shares more than 90% sequence identity.^11, 12^ Owing to these biological characteristics, these genes have similar biological function.^13^ FUT3, FUT5 and FUT6 are related to the occurrence and metastasis of gastric cancer (differential expression of *α*-2,3-sialyltransferases and *α*-1,3/4).^14, 15^ According to previous studies, high expression of FUT3 in CRC promotes metastasis.^8^ We hypothesised that FUT5 and FUT6 may promote proliferation, migration and invasion of CRC. In addition, according to our previous research, FUT can be regulated by miRNA in breast cancer.^16^ We further investigated whether FUT5 and FUT6 are regulated by miRNAs in CRC.

MicroRNAs are small (19–25 nt), non-coding, regulatory RNAs that can regulate a wide variety of genes^17^ via suppressing the expression of target genes.^18^ The importance of miRNA function in physiology and disease has been widely recognised. miRNAs are differentially expressed in normal and tumour tissues in many types of cancers, including CRC.^19, 20, 21^ miR-483 and miR-551 have been validated as anti-oncogenes of CRC.^22^ We suspect that there may be some new miRNAs that are differentially expressed and regulate FUT5 and FUT6 in CRC. We used public prediction algorithms, Targetscan and microRNA.org and initially identified FUT5 and FUT6 as potential miR-125a-3p targeted genes. qPCR, a dual-luciferase reporter assay and functional experiments were used to further verify that FUT5 and FUT6 are target genes for miR-125a-3p. According to our previous studies, FUT family expression markedly modulated activity of the PI3K/Akt pathway in human hepatocellular carcinoma.^23^ We investigated whether this abnormal activation occurs in CRC.

The PI3K/Akt pathway has a critical role in most of the hallmark properties of cancer, including proliferation, tumourigenesis, tumour growth and angiogenesis.^24, 25^ Several reports highlight that aberrant activation of PI3K-AKT can promote cancer invasion and metastasis in many tumours, including CRC.^26, 27^ Numerous negative regulators, including regulatory proteins and miRNAs, inhibit the PI3K/Akt pathway and function as tumour suppressors in CRC.^28^ However, little is known regarding the effects of the miR-125a-3p/FUT5-FUT6 axis on the PI3K/Akt pathway in CRC. In this study, we assessed whether the miR-125a-3p/FUT5-FUT6 axis had an effect on the PI3K/Akt pathway by western blot. Furthermore, we used LY294002 and Akt siRNA to investigate the effects of the PI3K/Akt pathway in CRC.

Therefore, the purpose of the present study was to identify miR-125a-3p as a new anti-oncogene, which regulates FUT5 and FUT6 and affects aberrant activation of the PI3K/Akt pathway in CRC. miR-125a-3p may serve as a predictive biomarker and a potential therapeutic target in CRC treatment.

## Results

**Figure 1:**
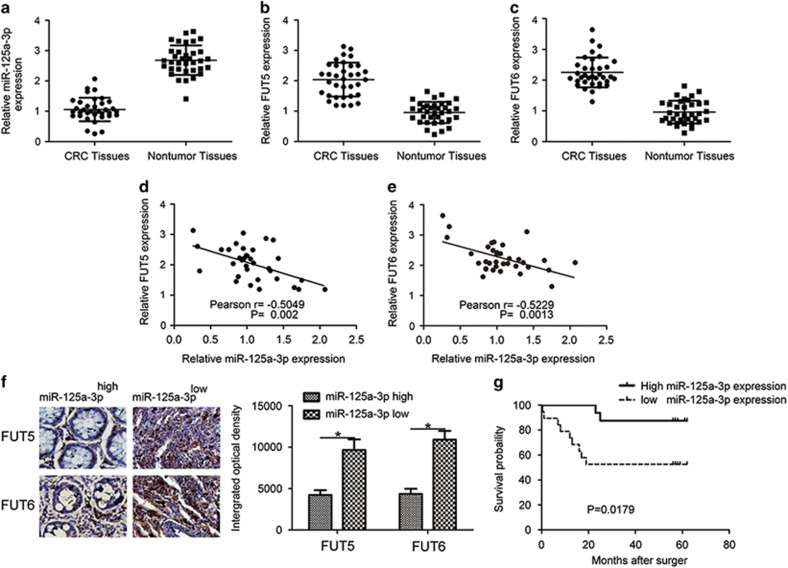
miR-125a-3p is inversely associated with FUT5 and FUT6 in CRC specimens. (**a**) The expression of miR-125a-3p was significantly downregulated, whereas (**b** and **c**) the expression of FUT5 and FUT6 was significantly upregulated in CRC tissues. (**d** and **e**) The inverse relationship between FUT5 or FUT6 expression and miR-125a-3p. (**f**) In tissues, a low expression of miR-125a-3p was observed, whereas FUT5 or FUT6 was high. (**g**) Kaplan–Meier overall survival curves according to miR-125a-3p expression. The overall survival of the high miR-125a-3p group was significantly higher than that of the low miR-125a-3p group (*P<0.05)

miR-125a-3p is inversely associated with FUT5 and FUT6 in CRC tissues. To investigate the correlation of FUT5 and FUT6 expression with miR-125a-3p in CRC tissues, we examined miR-125a-3p, FUT5 and FUT6 expression from 35 pairs of CRC patients by qPCR (Supplementary Table 1). The expression of miR-125a-3p was significantly decreased in CRC tissues compared with normal tissue (Figure 1a), whereas the expression levels of FUT5 and FUT6 were significantly upregulated in cancer tissue (Figures 1b and c). Through data analysis, we found an inverse relationship between FUT5 or FUT6 expression and miR-125a-3p in CRC tissues (Figures 1d and e). Correspondingly, immunostaining analysis of FUT5 or FUT6 was performed. In the tissues with low of miR-125a-3p, FUT5 and FUT6 expression was high (Figure 1f). With regard to overall survival, patients with low miR-125a-3p expression had a significantly poorer prognosis (Figure 1g). These results imply that low expression of miR-125a-3p and its associated expression of FUT5 and FUT6 may be related to CRC development.

**Figure 2:**
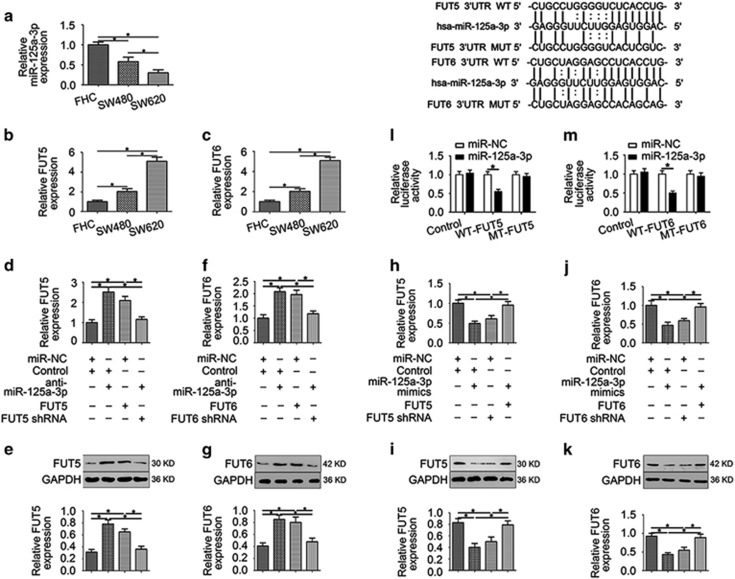
FUT5 and FUT6 are direct targets of miR-125a-3p. (**a**) miR-125a-3p expression was markedly reduced in SW620 cells. (**b** and **c**) In contrast, we observed both FUT5 and FUT6 overexpression in SW620 cells. (**d**–**g**) According to qPCR and western blot analysis, we found that anti-miR-125a-3p transfection led to significantly increased expression of FUT5 or FUT6 in SW480 cells (**h**–**k**), whereas miR-125a-3p-mimics led to decreased expression of FUT5 or FUT6 in SW620 cells. (**l** and **m**) Luciferase activity assay showed that FUT5 and FUT6 are direct targets of miR-125a-3p (*P<0.05)

FUT5 and FUT6 are direct targets of miR-125a-3p in CRC cells. On the basis of inverse relationship between FUT5 or FUT6 expression and miR-125a-3p in CRC tissues, we hypothesised that FUT5 and FUT6 may be direct targets of miR-125a-3p. First, we analysed the expression of miR-125a-3p via a miRNA microarray (data not shown) in SW480 (poorly invasive) and SW620 (highly invasive) cells. The expression of miR-125a-3p was low in SW620 cells compared with SW480 cells. Similar results were obtained by qPCR analysis, which showed that miR-125a-3p expression was markedly reduced in SW620 cells compared with SW480 and FHC (Figure 2a). In contrast, we observed that both FUT5 and FUT6 are overexpressed in SW620 cells compared with SW480 and FHC cells (Figures 2b and c).

To gain mechanistic insight into how FUT5 and FUT6 regulated by miR-125a-3p, we used public prediction algorithms, Targetscan and microRNA.org and initially identified FUT5 and FUT6 as potential miR-125a-3p targeted genes. To provide evidence for this prediction, qPCR was used to analyse FUT5 and FUT6 expression in SW480 cells transfected with anti-miR-125a-3p, FUT5 or FUT6. We found that anti-miR-125a-3p transfection led to a significant increase in FUT5 or FUT6 expression, which similarly to affected FUT5 or FUT6 regulation (Figures 2d and f). Furthermore, the effect of miR-125a-3p inhibitors can be reversed by transfection with FUT5 shRNA or FUT6 shRNA in SW480 cells. On the other hand, expression of FUT5 or FUT6 was blocked by overexpression of miR-125a-3p, which similar to effect of FUT5 shRNA or FUT6 shRNA in SW620 (Figures 2h and j). Moreover, western blot analysis also displayed similar results (Figures 2e, g, i and k).

Next, we a used dual-luciferase reporter assay to test whether FUT5 and FUT6 are direct targets of miR-125a-3p. The results showed that co-transfection of HEK293T cells with miR-125a-3p-mimics inhibits WT FUT5 3′-UTR regulated luciferase activity compared with the control but did not affect the luciferase activity of the MT FUT5 3′-UTR reporter (Figure 2l). Similarly, the luciferase activity assay showed that overexpression of miR-125a-3p significantly reduced the luciferase activity of WT FUT6 3′-UTR reporter plasmids, but had no effect on MT FUT6 3′-UTR reporters (Figure 2m).

Taken together, we conclude that both FUT5 and FUT6 are direct targets of miR-125a-3p.

### The miR-125a-3p/FUT5-FUT6 axis regulates cell proliferation and migration in CRC

**Figure 3:**
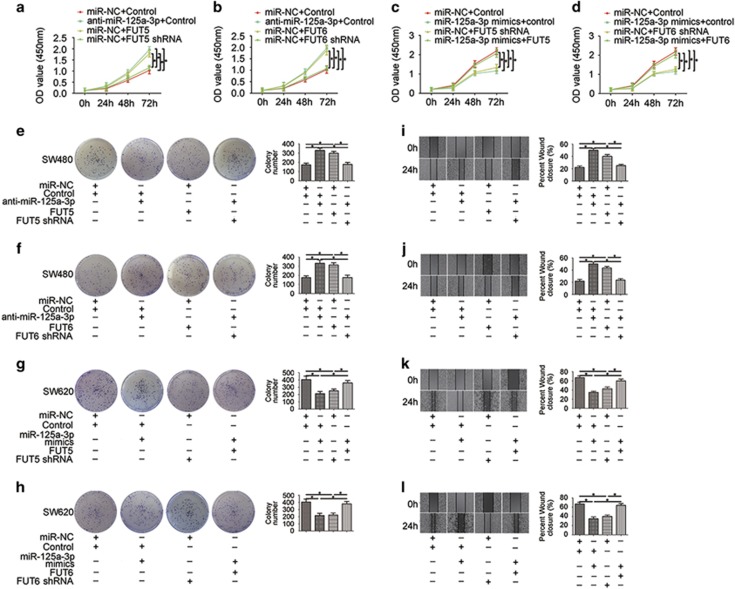
The miR-125a-3p/FUT5-FUT6 axis regulates the proliferation and migration ability of CRC cells. (**a**and **b**) The proliferation capacity was tested in SW480 cells transfected with anti-miR-125a-3p, FUT5 or FUT6 by CCK8 assays and (**e** and **f**) clone formation assays. (**c**, **d**,**g**and **h**) Proliferation capacity was downregulated in SW620 cells transfected with miR-125a-3p-mimics, FUT5 shRNA or FUT6 shRNA. (**i**–**l**) Wound healing assay was used to detect cell migration capacity (*P<0.05)

We investigated the role of the miR-125a-3p/FUT5-FUT6 axis in the proliferation of CRC cells by CCK8 assays and a colony-formation assay. In the CCK8 cell proliferation assay, we found that cell proliferation was promoted after miR-125a-3p knockdown or overexpression of FUT5 and FUT6 in SW480 cells (Figures 3a and b), whereas inhibition of FUT5 and FUT6 or overexpression of miR-125a-3p could suppress the proliferation of SW620 cells (Figures 3c and d). Furthermore, a colony-formation assay showed that SW480 cells transfected with anti-miR-125a-3p or FUT5 or FUT6 had a more efficient colony-formation rate (Figures 3e and f). Similar to the effects of SW620 cells transfected with miR-125a-3p-mimics, FUT5 shRNA or FUT6 shRNA impaired colony formation, and miR-125a-3p-dependent inhibition of FUT5 and FUT6 was rescued by reintroduction of FUT5 or FUT6 in SW620 cells (Figures 3g and h). To assess the influence of the miR-125a-3p/FUT5-FUT6 axis on cell migration, a wound healing assay was used to detect cell migration capacity via covering scratched areas. The data indicated that wound closure in the anti-miR-125a-3p, FUT5 or FUT6 group was significantly increased in SW480 cells (Figures 3i and j). Alternatively, miR-125a-3p-mimics or the FUT5 shRNA or FUT6 shRNA group exhibited reduced wound closure compared with the control group in SW620 cells (Figures 3k and l).

These results indicate that inhibition of miR-125a-3p or overexpression of FUT5 and FUT6 can promote proliferation and migration in CRC cells.

### The miR-125a-3p/FUT5-FUT6 axis regulates the invasion and angiogenesis of CRC cells

**Figure 4:**
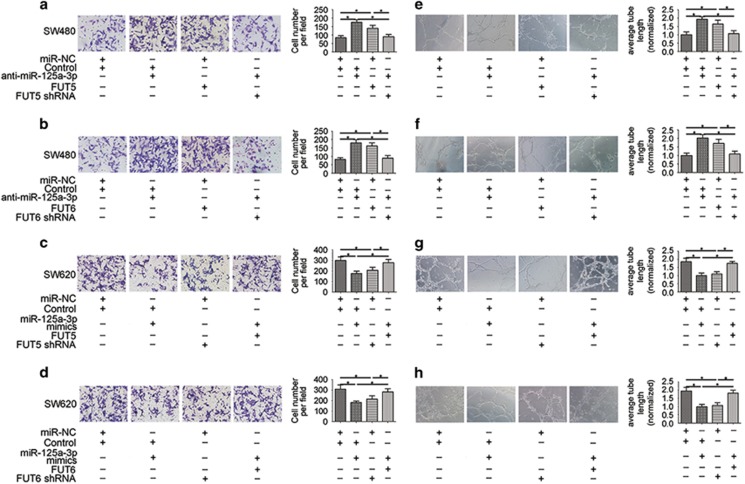
The miR-125a-3p/FUT5-FUT6 axis regulates the invasion and angiogenesis ability of CRC cells. (**a**and **b**) Transwell assays showed that SW480 cells transfected with anti-miR-125a-3p or FUT5 or FUT6, exhibited increased invasive ability. (**c** and **d**) The expression of miR-125a-3p-mimics, FUT5 shRNA or FUT6 shRNA reduced the invasion ability of SW620 cells. According to an endothelial tube formation assay, (**e** and **f**) the length of tubes was significantly increased in the anti-miR-125a-3p, FUT5 or FUT6 group, and (**g**and **h**) an opposite result was found with the miR-125a-3p-mimics, FUT5 shRNA or FUT6 shRNA group (*P<0.05)

Transwell assays were used to evaluate the contribution of the miR-125a-3p/FUT5-FUT6 axis in CRC cell invasion. The results showed that SW480 cells transfected with anti-miR-125a-3p or the FUT5 or FUT6 group had increased invasion compared with the control (Figures 4a and b), whereas the expression of miR-125a-3p-mimics, FUT5 shRNA or FUT6 shRNA reduced the invasion ability of SW620 cells (Figures 4c and d). New blood vessels are essential to promote tumour development. To assess the influence of the miR-125a-3p/FUT5-FUT6 axis in tumour development, we utilised an endothelial tube formation assay. The length of tubes was significantly increased by proangiogenic stimuli from the supernatant of the anti-miR-125a-3p, FUT5 or FUT6 group (Figures 4e and f), and opposing results were found in the miR-125a-3p-mimics, FUT5 shRNA or FUT6 shRNA group (Figures 4g and h).

These data suggest that overexpression of FUT5 or FUT6 induced by miR-125a-3p promoted the invasion ability of CRC cells and development of tumours.

### The miR-125a-3p/FUT5-FUT6 axis regulates CRC cell growth *in vivo*

**Figure 5:**
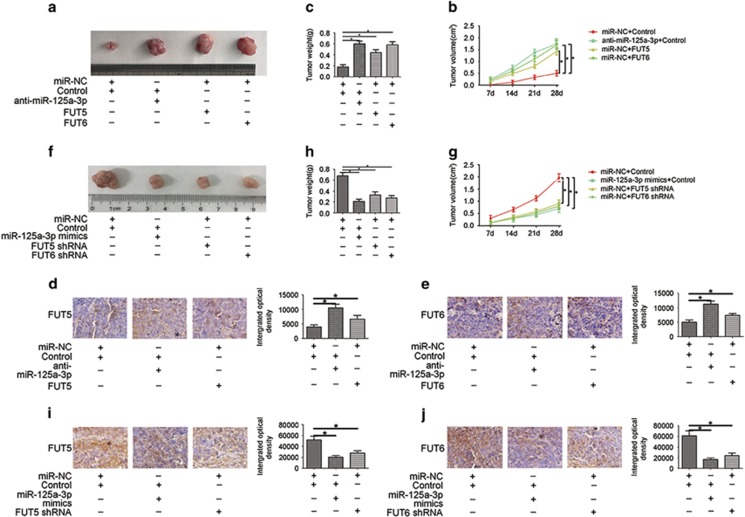
The miR-125a-3p/FUT5-FUT6 axis regulates CRC cell growth *in vivo*. (**a**and **b**) Tumour growth curves were measured after injection of SW480 cells transfected with anti-miR-125a-3p, FUT5 or FUT6 and (**f** and **g**) SW620 cells transfected with miR-125a-3p-mimics, FUT5 shRNA or FUT6 shRNA. (**c**and **h**) Tumour weights were measured after the tumours were removed. Immunofluorescence staining assay with FUT5 or FUT6 antibodies was used to assess proliferation capacity in (**d** and **e**) SW480 and (**i**and **j**) SW620 cells (*P<0.05)

On the basis of the results of functional studies *in vitro*, we further established xenografts to evaluate CRC cell growth activity. For this, miR-NC/control, SW480/FUT5, SW480/FUT6, SW620/ FUT5 shRNA and SW620/ FUT6 shRNA cells were injected into nude mice. We simultaneously, we used a miRNA delivery method to increase or reduce miR-125a-3p in tumour cells of mouse xenografts. After 4 weeks, the growth of tumours transfected with anti-miR-125a-3p, SW480/FUT5, and SW480/FUT6 significantly increased compared to the control (Figures 5a–c), whereas tumour growth was significantly decreased in the nude mice transfected with miR-125a-3p-mimics, SW620/FUT5 shRNA or SW620/FUT6 shRNA (Figures 5f–h). Correspondingly, immunostaining analysis of FUT5 or FUT6 was performed in harvested tumour tissues. In SW480 xenograft tumours, FUT5 or FUT6 protein increased in the SW480/FUT5 or SW480/FUT6 group and anti-miR-125a-3p group (Figures 5d and e). In SW620 xenograft tumours, miR-125a-3p overexpressing tumours showed low FUT5 and FUT6 protein levels, and FUT5 or FUT6 protein reduced in the SW620/FUT5 shRNA or SW620/FUT6 shRNA group (Supplementary Figure 1; Figures 5i and j).

Altogether, these findings confirmed that FUT5 and FUT6 possess tumour stimulating activities in CRC tumours, which was regulated by miR-125a-3p.

### The miR-125a-3p/FUT5-FUT6 axis mediates activity of the PI3K/Akt signalling pathway in CRC cells

**Figure 6:**
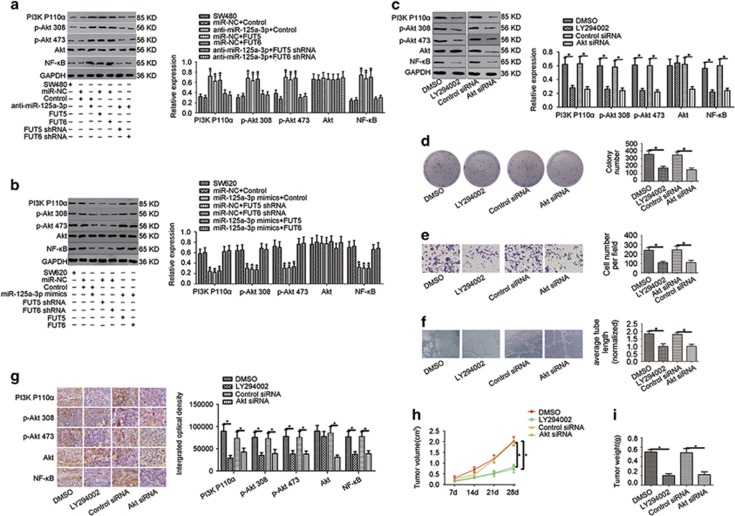
The miR-125a-3p/FUT5-FUT6 axis mediates the activity of the PI3K/Akt signalling pathway. (**a**) In SW480 cells transfected with anti-miR-125a-3p, FUT5 or FUT6, PI3K p110*α*, Thr308, Ser473 and NF-kB were greatly increased, and (**b**) an opposite result was found in SW620 cells transfected with miR-125a-3p-mimics, FUT5 shRNA or FUT6 shRNA. (**c**) In addition, western blotting showed that PI3K110*α*, Akt Thr308, Akt Ser473, Akt and NF-kB were decreased by LY294002 treatment or Akt siRNA in SW620 cells. The results of the (**d**) colony-formation assay, (**e**) transwell invasion assay, and (**f**) endothelial tube formation assay, showed that proliferation, invasion and angiogenesis abilities of SW620 cells were reduced by LY294002 treatment or Akt siRNA. Tumour growth was significantly decreased in nude mice given by LY294002 treatment or transfected with Akt siRNA based on (**g**) immunostaining, (**h**) tumour growth curves and (**i**) tumour weight (*P<0.05)

We previously reported that FUT6 enhanced PI3K kinase activity in human hepatocellular carcinoma. Therefore, we tested the influence of the miR-125a-3p/FUT5-FUT6 axis on activation of the PI3K/Akt signalling pathway by the western blot. Western blot showed that PI3K p110a or phosphorylation of Akt at Thr308 and Ser473 and NF-kB were greatly enhanced in SW480 treated with FUT5, FUT6 or anti-miR-125a-3p. In contrast, the total amount of Akt protein remained unchanged (Figure 6a). In addition, PI3K110a, Akt Thr308, Akt Ser473 and NF-kB in SW620 cells transfected with miR-125a-3p-mimics, FUT5 shRNA or FUT6 shRNA were significantly reduced (Figure 6b). The effect of anti-miR-125a-3p or miR-125a-3p-mimics was rescued by FUT5 and FUT6 or FUT5 shRNA and FUT6 shRNA, respectively. Collectively, these results suggest that the miR-125a-3p/FUT5-FUT6 axis affected the PI3K/Akt pathway.

To further estimate the effect of the PI3K pathway on FUT5 and FUT6 overexpressing cells, SW620 cells were treated with a PI3K/Akt targeted inhibitor or Akt siRNA. Western blotting confirmed that PI3K110a, Akt Thr308, Akt Ser473 and NF-kB were decreased by LY294002 treatment or Akt siRNA (Figure 6c). Next, we investigated the role of PI3K/Akt pathways by colony-formation assay, transwell assay and endothelial tube formation assay in SW620 cells. As expected, both LY294002 treatment and Akt siRNA reduced the proliferation, invasion and angiogenesis ability of SW620 cells (Figures 6d–f). Similar results were also observed in tumourigenicity analysis *in vivo*. Reduced tumour growth and weight were measured in mice bearing SW620 tumours with an impaired the PI3K/Akt signalling pathway (Figures 6h and i). Correspondingly, immunostaining analysis of PI3K110a, Akt Thr308, Akt Ser473, Akt and NF-kB were performed in harvested tumour tissues, displaying similar results as western blotting in that PI3K110a, Akt Thr308, Akt Ser473 and NF-kB were decreased by LY294002 treatment or Akt siRNA (Figure 6g). These data further suggested that the proliferation, invasion and angiogenesis ability of SW620 cells were associated with the PI3K/AKT pathway activity.

## Discussion

Colorectal cancer is a disease characterised by high morbidity and mortality. In this study, we investigated whether miR-125a-3p has an inhibitory effect on CRC via targeting both FUT5 and FUT6. We found that the miR-125a-3p/FUT5-FUT6 axis mediated the PI3K/Akt signalling pathway, which regulated the proliferation, invasion and angiogenesis ability of CRC cells. We showed that (1) both FUT5 and FUT6 were highly expressed in CRC tissues and cell lines, which enhanced the proliferation, migration, invasion and angiogenesis capacity of CRC cells and tumour growth *in vivo*, and (2) miR-125a-3p was significantly downregulated in CRC tissues and cell lines, as miR-125a-3p expression could greatly inhibit migration, invasion and angiogenesis of CRC cells and tumour growth *in vivo*, further improving survival. Furthermore, miR-125a-3p was inversely correlated with FUT5 and FUT6 expression, which dramatically attenuated effect of FUT5 and FUT6. (3) In addition, miR-125a-3p, FUT5 or FUT6 expression markedly modulated the activity of the PI3K/Akt signalling pathway, whereas the effect of FUT5 or FUT6 can be reversed by transfection with miR-125a-3p-mimics. In summary, our work suggests that miR-125a-3p is a potent anti-oncogene of CRC.

In recent years, numerous studies have shown that miRNAs could serve functionally as ‘oncogenes^29^’ or ‘tumour suppressor genes^30^’ in the genesis of a variety of cancers, including CRC.^31^ The miR-125a family suppresses tumour growth in renal cell carcinoma^32^ and breast tumourigenesis,^33^ and is increasingly recognised as an important anti-oncogene. Consistently, miR-125a-3p and miR-125a-5p inhibit the invasion and migration of lung cancer cells.^34^ Moreover, miR-125a-5p inhibits cell proliferation in CRC.^35^ We hypothesised that miR-125a-3p may also have similar effects in CRC. In this study, we found that miR-125a-3p expression was significantly decreased in CRC tissues by qPCR analysis. Next, we investigated the role of miR-125a-3p in CRC. *In vitro*, using functional analyses, we found that overexpression of miR-125a-3p inhibited cell proliferation migration, invasion and development of CRC cells. *In vivo*, overexpression of miR-125a-3p inhibited tumour growth. Our data suggest that miR-125a-3p may be an important anti-oncogene in CRC.

Aberrant expression of fucosylated N-glycans has been observed in a variety of human malignancies. These N-glycans are synthesised by FUTs. In our previous study, the FUT family was found to be abnormally expressed in breast cancer^16^ and hepatocellular carcinoma.^36^ Several studies clearly demonstrated that FUT6 expression was high in CRC.^37, 38^ Likewise, we showed, for the first time, that both FUT5 and FUT6 were upregulated in CRC. In this experiment, our gain- and loss-of-function analyses showed that altered FUT5 and FUT6 regulated cell proliferation, migration, and invasion *in vitro* and tumour growth *in vivo*. Then, we further investigated whether there is a relationship between elevation of FUT5 and FUT6 and decrease of miR-125a-3p. According to our previous research, FUT can be regulated by miRNA. We hypothesised that FUT5 and FUT6 may be target genes of miRNA-125a-3p.

Recently, more attention has been paid to the detection of miRNAs and their target genes. For example, BCL2, BCL2L12 and MCL1 are target genes of miR-125a-5p in CRC.^35^ miRNA-125a-3p reduces cell proliferation and migration by targeting Fyn associated with several types of cancer.^39^ On the basis of our previous research, FUT can be regulated by miRNAs and influence the proliferation and invasion of cancer cells. To search for miRNAs targeting FUT5 and FUT6, we analysed bioinformatics and conducted a dual-luciferase assay. Combined with results of the bioinformatics analysis and luciferase activity assays, both FUT5 and FUT6 were identified as target genes of miR-125a-3p. Furthermore, FUT5 and FUT6 overexpression dramatically attenuated the effect of miR-125a-3p, whereas this effect of FUT5 or FUT6 can be reversed by transfection with miR-125a-3p-mimics. In summary, FUT5 and FU6 were found to be novel direct targets of miR-125a-3p.

The PI3K pathway controls proliferation, invasion and angiogenesis^40^ in many tumours, including CRC. In addition, PI3K-pathway activation occurs concomitantly with RAS/BRAF mutations in CRC.^41^ Furthermore, understanding the PI3K pathway will lead to more effective treatments and biomarker identification in CRC patients.^42, 43^ In our previous report, altered expression of FUT6 markedly modulated the activity of the PI3K/Akt pathway in human hepatocellular carcinoma cell lines.^44^ However, the report did not discuss the PI3K/Akt pathway as a downstream target of FUT5. In this study, we investigated whether the miR-125a-3p/FUT5-FUT6 axis mediated the PI3K/Akt signalling pathway in CRC. To test the effects of the miR-125a-3p/FUT5-FUT6 axis on PI3K/Akt pathway, we used western blot analysis. The results demonstrated that the miR-125a-3p/FUT5-FUT6 axis markedly effects Akt phosphorylation. In addition, the proliferation, invasion and angiogenesis abilities of SW620 cells were reduced when the PI3K/Akt pathway was inhibited. Hence, our findings revealed that the miR-125a-3p/FUT5-FUT6 axis was involved in PI3K/Akt pathway activation, which regulates the proliferation, invasion and angiogenesis ability of CRC cells. However, further investigations are still needed to explore whether the miR-125a-3p/FUT5-FUT6 axis can affect RAS/BRAF mutations, which occur concomitantly with PI3K-pathway activation in CRC.

In conclusion, our study demonstrated that overexpression of miR-125a-3p attenuated the migration, invasion and angiogenesis of CRC cell lines and inhibited tumour growth *in vivo* by affecting FUT5 or FUT6 regulated expression through the PI3K/Akt signalling pathway. miR-125a-3p may represent a novel strategy with biological significance and diagnostic and prognostic value.

## Materials and methods

### Tissue samples

Human CRC tissues were collected from 35 patients, obtained with informed consent and in accordance with the ethical standards of the Second Hospital of Dalian Medical University (Dalian, China) Review Board. The patients included 17 men and 18 women, with ages ranging from 28 to 85 years (mean age of 49.8 years). No patients had received chemotherapy or radiation therapy. The patient tissues were snapfrozen in liquid nitrogen and stored at −80 °C until RNA extraction.

### Cell culture

Human normal colorectal epithelial cell line (FHC) and CRC cell line, including SW480 and SW620, cells were obtained from KeyGEN Company (Nanjing city, Jiangsu Province, China). Human embryonic kidney cell line (HEK293T) cells and umbilical vein endothelial cells (HUVECs) were obtained from the Institute of Biochemistry (Shanghai, China). FHC cells, HEK293T cells and HUVECs were cultured in 90% DMEM (Gibco) supplemented with antibiotics (1 × penicillin/streptomycin100 U/ml, Gibco) and 10% heat-inactivated foetal bovine serum (FBS) (Gibco, Grand Island, NY, USA). SW480 and SW620 cells were cultured in 90% L-15 (Gibco) supplemented with antibiotics and 10% FBS. The cells were incubated at 37 °C in a humidified and 5% CO_2_ incubator.

### PCR analysis

RNA extraction, including miRNA extraction, from cell lines and frozen tissues was performed by TRIZOL reagent, and cDNA was synthesised using the QuantiTect Reverse Transcription Kit (Qiagen, Valencia, CA, USA) according to the manufacturer’s protocol. FUT mRNA was quantified by SYBR-Green qPCR (Takara, Otsu, Shiga, Japan) and normalised to GAPDH. The expression of mature miR-125a-3p was determined by qPCR with the mirVanaTM qPCR microRNA Detection Kit (Ambion, Austin, TX, USA) according to manufacturer’s protocol and relative to U6-small nuclear RNA. The sequences of upstream and downstream primers were as follows: miR-125a-3p, 5′-ACACTCCAGCTGGGACAGGTGAGGTTCTTG-3′ and 5′-CTCAACTGGTGTCGTGGAGTCGGCAATTCAGTTGAGGGCTCCCA-3′, respectively; U6, 5′-CTCGCTTCGGCAGCACA-3′ and 5′-AACGCTTCACGAATTTGCGT-3′, respectively; FUT5, 5′-ATGGCAGTGGAACCTGTC-3′ and 5′-GCACCATCTCTGAGCAGC-3′,respectively; FUT6, 5′-CATTTCTGCTGCCTCAGG-3′ and 5′-GGGCAAGTCAGGCAACTC-3′, respectively; GAPDH, 5′-CTCCTCCACCTTTGACGCTG-3′ and 5′-TCCTCTTGTGCTCTTGCTGG-3′, respectively. All PCR reactions were performed in triplicate.

### Western blot analysis

Whole cell proteins were electrophoresed under reducing conditions in 10% polyacrylamide gels. The electrophoresis was run in MOPS buffer at 180 V for 1 h. After blocking in 5% nonfat dry milk, the membrane was incubated with antibody (Abcam, Cambridge, UK, 1:1000 dilution) overnight at 4 °C. All band intensities were evaluated using an ECL western blotting kit (Amersham Biosciences, Little Chalfont, UK) according to the manufacturer’s instructions, and the results were analysed with Image-J software.

### Deregulation of FUT5 or FUT6 in SW620 cells by RNAi

For plasmid transfection, 1.5 × 10^5^ SW620 cells were implanted and cultured in a 12-well plate for 24 h. SW620 cells were cultured in 1 ml of complete medium with 5 mg/ml polybrene (sc-134220, Santa Cruz Biotech) per well and treated with 0.4 *μ*M FUT5 or FUT6 specific shRNA lentiviral particles (sc-40616- V and sc-72405- V, Santa Cruz Biotech) overnight, and three control wells were transfected with control shRNA lentiviral particles (sc-108080, Santa Cruz Biotech, TX, USA). The sequences of siRNAs primers were as follows: FUT5 shRNA, 5′-GCTTATGGCAGTGGAACCTGT-3′, and FUT6 shRNA, 5′- GTCTCAAGACGATCCCACTGT-3′. The transfection efficiency was approximately 81%, and cell viability was 85%. Forty-eight hours post infection, the cells were collected and processed for various assays.

### Overexpression of FUT5 or FUT6 in SW480 cells

The human FUT5 and FUT6 coding sequences were purchased from TaKaRa company (Dalian, China) and were transfected into the pEGFP-N2 vector (Invitrogen, Carlsbad, CA, USA), using *EcoR*I and XhoI sites. After 4 weeks of screening, cell lines stably expressing FUT5 (SW480/FUT5), FUT6 (SW480/FUT6) and empty vector (SW480/mock) were established. The cell transfection efficiency was 80%, and the survival rate was 82%.Then, the cells were collected for gene expression assays and further study.

### Transfection assay

miR-125a-3p-mimics, negative control oligonucleotides (miR-NC) and miR-125a-3p inhibitors (anti-miR-125a-3p) were purchased from RiboBio (Guangzhou, China). miR-125a-3p-mimics were transfected into SW620 cells, and anti-miR-125a-3p was transfected into SW480 cells. The tumour cells (5 × 10^3^cells per well) were cultured in a 24-well dish. The transfection was performed using Lipofectamine 2000 reagent (Invitrogen) according to the manufacturer’s instructions. The sequences of miR-125a-3p inhibitor primers were as follows: 5′-GGCUCCCAAGAACCUCACCUGU-3′. Forty-eight hours post infection, cells were collected and processed for various assays, and fluorescence microscopy was performed to assess the efficiency of transfection efficiency. The transfection efficiency was ~80%.

### IHC staining analysis

Paraffin-embedded tumour blocks were sectioned, dried overnight at 37 °C, then deparaffinized with xylene and rehydrated. After deparaffinization and pretreatment with 3% hydrogen peroxide prior to antibody incubation, the slides were labelled overnight at 4 °C with antibodies (Abcam). Secondary antibody binding (allDako, Santa Clara, CA, USA, 1:200) was visualised using a streptavidin ABC kit (Santa Cruz Biotech) at 37 °C for 1 h, and positive staining was visualised with DAB substrate chromogen solution. Finally, the sections were counterstained with haematoxylin and cover slipped.

### Dual-luciferase reporter assays

Luciferase assays were conducted in HEK293T cells. FUT5 and FUT6 wild-type (WT) sequences of the 3′-UTR were cloned downstream of the firefly luciferase gene in the pGL3-control vector (Promega, Madison, WI, USA), and mutant (MT) 3′-UTR plasmid mutations were performed using the QuickChange XL site-directed mutagenesis kit (Stratagene, Agilent Technologies, Santa Clara, CA, USA). Mimics and control oligonucleotides for miR-125a-3p were obtained from RiboBio (GenePharma, Shanghai, China). HEK293T cells were plated in (5 × 10^4^ cells per well) a 12-well dish and incubated overnight. miR-125a-3p-mimics and WT or mutant target sequence were co-transfected into HEK293T cells via Lipofectamine 2000. Plates were incubated at 37 °C for 48 h. Luciferase activities were determined with the Dual-Luciferase R Reporter Assay System (Promega, Madison, WI, USA) and normalised to Renilla luciferase activities. The mean of the results from cells with miR-NC was set at 1.0. Luciferase assays were performed in triplicate.

### Cell proliferation assays

Cell Counting Kit-8 (CCK8) (Biotool, Houston, TX, USA) was used as a quantitative endpoint to assess the proliferation ability of SW480 and SW620 cells. Cells were plated in 96-well plates at 2 × 10^3^ cells per well containing complete L-15 in triplicate for each condition. The CCK8 solution was added to each well and incubated for 4 h. Then, OD was measured by a WST (water-soluble tetrazolium salt) assay according to the CCK8 assay kit protocol (Biotool, Houston, TX, USA). The absorbance of each well was quantified at 450 nm on a microplate reader (168–1000 Model 680, Bio-Rad, Hercules, CA, USA).

### Colony-formation assay

Cells were seeded into 6-well plates at 1 × 10^3^cells per plate. The cells were mixed and then cultured for 10 days with culture medium containing L-15 with 10% FBS. The following criteria were considered for evaluating the results: clusters of ⩾30 cells were counted as a colony.

### Wound healing assay

Tumour cells (4 × 10^5^cells per well) were seeded into a 12-well plate. A wound was created by scraping monolayer cells with a sterile pipette tip across the monolayer when adherent cells were observed after 24 h. Next, the migration path of cells was tracked at 0 h and 24 h using Olympus microscope (10 × 10), and representative scratch zones for each cell line were photographed. The results of experiments were analysed by the software ipp6.0 (Media Cybernetics, Bethesda, MD, USA).

### Transwell invasion assay

Cell invasion assay was performed using Boyden chambers containing a transwell membrane filter (Corning, New York, NY, USA). Cells were serum starved overnight, harvested, and resuspended in migration medium (L-15 medium with 0.5% BSA). Then, a suspension of 4 × 10^5^ cells in 100 *μ*l of migration medium was seeded on top. L-15 with 10% FBS was placed in the lower compartment of the chamber as a chemo-attractant. After 24 h of incubation, cells on the upper side were removed with a cotton swab. Evaluation of invasive capacity was performed by counting invading cells under a microscope (40 × 10). Five random fields of view were analysed for each chamber.

### Endothelial tube formation assay

Matrigel (Corning) (50 *μ*l) was added to each well of a 96-well plate and was allowed to solidify (37 °C, 30 min). HUVECs were resuspended in the supernants collected from the RNAi group, RNA group and control groups. Then, 300 *μ*l of supernatants was added to each well containing 4 × 10^4^ HUVECs and incubated at 37 °C and 5% CO_2_. After 8 h, tube formation was observed under a microscope, and the honeycomb-like tubular structures were quantified.

### Tumourigenicity assay *in vivo*

For the tumourigenicity assay, 1 × 10^6^ tumour cells in 0.1 ml of phosphate-buffered saline were injected subcutaneously into the right flank of nude mice (6 weeks old) obtained from the Animal Facility of Dalian Medical University; the mice, were fed sterilised food and water. Once palpable tumours were observed (~4 weeks after tumour cell inoculation), the animals were killed, and tumours were excised. All of the animal experiments were approved by the responsible governmental animal ethics committee.

### Inhibition of PI3K/Akt signalling

LY294002 (Sigma, Missouri, USA) was used to suppress the activity of PI3K/Akt signalling in SW620 cells. Briefly, SW620 cells (1 × 10^4^ cells per well) were incubated with DMSO (Sigma, St. Louis, MO, USA) or LY294002 and collected after 24 h. In addition, Akt expression was also silenced by RNAi. The sequences of AKT siRNA primers were as follows: 5′-GCACCTTTATTGGCTACAA-3′. Changes in protein expression were measured by western blot analysis. The proliferation, invasion and angiogenesis abilities of SW620 cells were measured by transwell assays, colony-formation assay and endothelial tube formation assay *in vitro* and tumorigenicity assay *in vivo*.

### Statistical analysis

The data were presented as the mean standard deviation (S.D.) from triple tests of each group. Student’s *t*-test was used to compare values between the test and control groups. Statistical significance was defined as *P*<0.05. All calculations were performed using SPSS software version 13.0 (SPSS Inc., Chicago, IL, USA).
