# Supplementary figures and images for: miR-125a-3p/FUT5-FUT6 axis mediates colorectal cancer cell proliferation, migration, invasion and pathological angiogenesis via PI3K-Akt pathway
Source: Cell Death Dis. 2017 Aug 3;8(8):e2968–. doi: 10.1038/cddis.2017.352 (PMC5596543; doi:10.1038/cddis.2017.352)

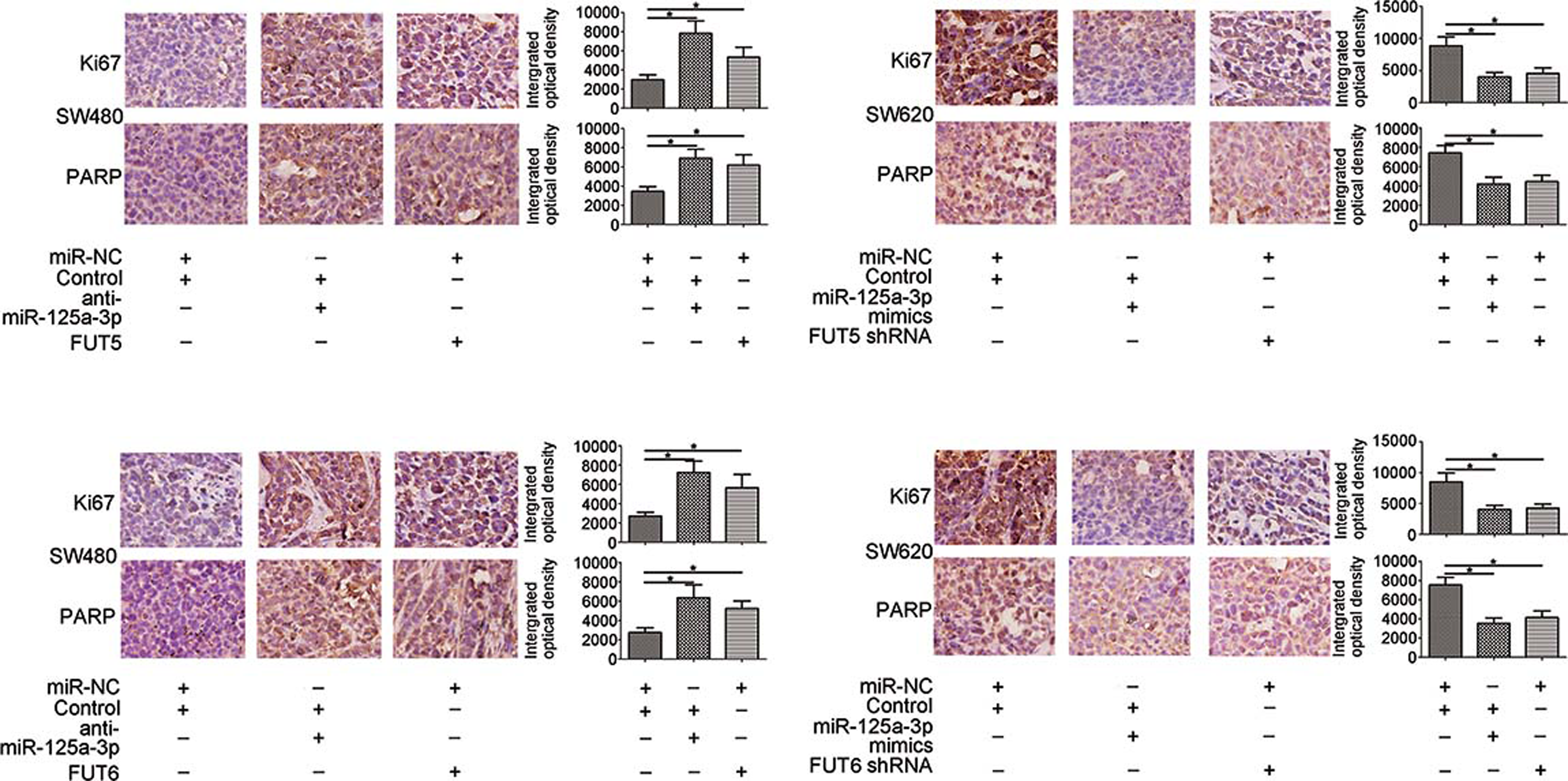

Supplement: Supplementary Data 1 [file cddis2017352x1.tif]
