# Supplementary material for: miR-125a-3p/FUT5-FUT6 axis mediates colorectal cancer cell proliferation, migration, invasion and pathological angiogenesis via PI3K-Akt pathway
Source: Cell Death Dis. 2017 Aug 3;8(8):e2968–. doi: 10.1038/cddis.2017.352 (PMC5596543; doi:10.1038/cddis.2017.352)
Supplement: Supplementary Information [file cddis2017352x3.docx]

Supplementary Figure 1 is placed behind the “In SW620 xenograft tumours, miR-125a-3p overexpressing tumours showed low FUT5 and FUT6 protein levels, and FUT5 or FUT6 protein reduced in the SW620/FUT5 shRNA or SW620/FUT6 shRNA group” in the **Results of** **“The miR-125a-3p/FUT5-FUT6 axis regulates** **CRC cell growth in vivo”.**( lines 188-190)

Supplementary Table 1 is placed behind the “To investigate the correlation of FUT5 and FUT6 expression with miR-125a-3p in CRC tissues, we examined miR-125a-3p, FUT5 and FUT6 expression from 35 pairs of CRC patients by qPCR” in the **Results of “**miR-125a-3p is inversely associated with FUT5 and FUT6 in CRC tissues**”.** ( lines 94-97)

Legends

Supplementary Figure 1

Immunofluorescence staining assay with Ki67 or PARP antibodies was used to assess proliferation capacity or apoptosis in SW480 and SW620 cells. Data show that Ki67 protein increased in the SW480 cells transfected with anti-miR-125a-3p, FUT5 or FUT6. PARP is similar to Ki67. (*P < 0.05)

Supplementary Table 1

The form shows specific information for 35 patients.
